# Social Affiliation and Attention to Angry Faces in Children: Evidence for the Contributing Role of Parental Sensory Processing Sensitivity

**DOI:** 10.3390/children12040524

**Published:** 2025-04-18

**Authors:** Antonios I. Christou, Kostas Fanti, Ioannis Mavrommatis, Georgia Soursou, Pantelis Pergantis, Athanasios Drigas

**Affiliations:** 1 Department of Special Education, University of Thessaly, 382 21 Volos, Greece; 2 Department of Psychology, University of Cyprus, Nicosia 1678, Cyprus; kfanti@ucy.ac.cy (K.F.); mavrommatis.ioannis@ucy.ac.cy (I.M.); soursou.georgia@ucy.ac.cy (G.S.); 3 Net Media Lab & Mind & Brain R&D, Institute of Informatics & Telecommunications, National Centre of Scientific Research ‘Demokritos’, 153 41 Agia Paraskevi, Greece; pantperg@helit.duth.gr (P.P.); dr@iit.demokritos.gr (A.D.); 4 Department of Information & Communication Systems Engineering, University of the Aegean, 832 00 Karlovasi, Greece

**Keywords:** eye tracking, emotion regulation, childhood, sensory processing sensitivity, social affiliation

## Abstract

Low social affiliation has been described as a phenotypic marker of antisocial behaviors by disrupting children’s initiation and enjoyment of positive physical and emotional connections with others. Laboratory studies have shown that, in early childhood, low social affiliation rates are preceded by lower attention to faces. In addition, while low social affiliation has also been associated with behavioral outcomes when accounting for parenting influences, the effect that parental environmental sensitivity may have on contributing to the link between emotional processing early in life and later behavioral manifestations associated with antisocial behavior is currently unknown. Objectives: The present eye-tracking study aims to delineate the potential contribution of parental Sensory Processing Sensitivity (SPS) to the link between children’s rates of social affiliation and their attentional patterns in response to emotional faces. Methods: For the needs of this study, children performed a lab-based, computerized facial processing task (N = 153; *Mage* = 9.97; *SDage* = 1.28; 48.8% females). In addition, one of the children’s parents completed questionnaires for their children’s and their own behavior (N = 153; *Mage* = 40.9; *SDage* = 4.85; 87.7% females). Results: The results underline the contributing role of parental SPS on the associations between social affiliation and visual scanning when viewing angry emotional faces. In particular, children with low rates of social affiliation spent significantly more time fixating on the mouth regions of angry faces only when their parents had higher SPS. Conclusions: This study unveils the possibility for the contextual influence of parental environmental sensitivity on the early emotional processing mechanisms in children with low rates of social affiliation. These findings suggest that interventions aimed at improving emotional processing in children at risk for antisocial behavior should consider parental SPS as a contributing factor. Tailoring parent-based interventions to address how highly sensitive parents interpret and respond to emotional challenges may reduce children’s attentional biases to threats and support healthier socioemotional development.

## 1. Introduction

Social affiliation describes the intrinsic motivation for the enjoyment of social bonding and positive emotional engagement with others in an individual’s environment [1]. Such behaviors are documented with the presence of a range of manifestations that promote interpersonal connection, such as social approach and social touch, positive vocalizations, and body gestures [1]. Social affiliation has been deemed critical for the support of mother–child bonding early in life [2], and in early childhood, it assists the development of social communication [3]. Conversely, the presence of low social affiliation and reduced sensitivity to cues of affiliation has been linked with difficulties in attachment formation in infancy [4] and problems with social bonding in childhood [5]. Interestingly, low social affiliation has been described as a phenotypic marker of callous–unemotional behaviors (i.e., CU traits [6]), which can inherently lead to an increased risk for antisocial behaviors [4,6]. In particular, low social affiliation is thought to increase the risk for CU traits by affecting children’s ability to make positive physical and emotional connections with significant others, leading to severe conduct problems [4,6].

### 1.1. Social Affiliation and Parenting

There is a plethora of evidence on facial emotion reactivity in relation to rates of social affiliation early in life, predominantly from adolescent and adult samples. More specifically, previous studies have shown that low social affiliation rates in early childhood are associated with lower attention to faces and reduced eye contact [7,8]. Interestingly, low social affiliation has been associated with behavioral outcomes when accounting for parenting influences [8]. Based on this account, the low arousal and hyporeactivity of neural and physiological systems may interact with caregiving environments characterized by harshness or a lack of affiliation that can lead to the manifestation of the behavioral phenotype of CU traits (e.g., [9,10]).

In particular, while parental warmth has been associated with reductions in CU behaviors between the ages of 2 and 3 [11] and reduced conduct problems (CPs) between the ages of 4 and 12 [12], harsh parenting has been linked to the increased desensitization of children to cues of threats or negative reinforcement (e.g., [13,14]). This later contextual influence may exacerbate the risk for aggression and CU behaviors among children with low fearful arousal (e.g., [14]). In sum, the current understanding in the literature suggests that parenting can be counted as an important environmental factor for the development of CU traits, while the temperament characteristics associated with CU traits (i.e., fearlessness or low affiliation) may undermine the parent–child relationship, which, in turn, can lead to more harsh and less warm parenting styles in a bidirectional manner (for a review, see [6]). Beyond parenting practices, it is currently unknown how individual differences in parental environmental sensitivity may lead to differing neurocognitive and exacerbating antisocial behavioral outcomes in children, which are related to CU traits.

### 1.2. Parenting and Sensory Processing Sensitivity

Sensory Processing Sensitivity (SPS) has been well described as a potential susceptibility marker [15] and is considered a reliable measure of the phenotypic trait of environmental sensitivity [16,17]. SPS has been associated with, among other factors, behavioral inhibition in unfamiliar situations, effortful control, and both negative and positive emotionality [18]. The literature has proposed a classification system that groups individuals into categories of “high”, “medium”, and “low” sensitivity, with high SPS characterized by deeper information processing, heightened awareness of environmental subtleties, and greater susceptibility to overstimulation (e.g., [18]). In adults, SPS is assessed using the Highly Sensitive Person Scale (HSPS) [19]. In its original description, individuals who are characterized with high SPS are more emotionally sensitive to both negative and positive environmental influences, leading to more intense impacts—positive and negative—on their personality and affectivity [19,20,21].

Despite the increasing evidence on the social–emotional characteristics of HSPs, we currently hold very limited knowledge on how individuals high in SPS may affect their children’s emotionality, and there is none regarding children’s neurocognitive responses to emotional information. In one study, it was documented that high-SPS mothers were more overwhelmed by their parenting responsibilities and tended to adopt more harsh parenting behavior, in addition to preferring spending time alone, withdrawing from the child, giving less focus and attention to the child’s needs, and showing more permissive parenting behavior [22]. Such behaviors may be perceived by children as inconsistent parenting, which may have a detrimental impact on the child’s social and emotional functioning.

Expanding upon this, Aron et al. [23] found that although high-SPS mothers reported experiencing parenting as more challenging, they also demonstrated greater emotional attunement with their children. Neurophysiological evidence supports this duality, showing that individuals with high Sensory Processing Sensitivity (SPS) exhibit increased brain activation in areas related to awareness, empathy, and self–other processing when exposed to emotional stimuli [24]. This activation is especially pronounced in response to close social partners (e.g., romantic partners), and particularly to positive emotional expressions. Extrapolating these findings to high-SPS parents, such individuals may display an intensified sensitivity to their child’s emotional cues, leading to increased awareness and the potential for empathetic responsiveness. However, this same heightened sensitivity may also render them more vulnerable to emotional overwhelm, which could, under stress or repeated strain, result in inconsistent or ineffective parenting practices. These dynamics are particularly relevant in the context of emerging evidence suggesting that negative or emotionally dysregulated parenting can shape early affective and attentional processes in children, especially those at risk for developing callous–unemotional (CU) traits [14]. Despite these theoretical connections, little is known about how parental environmental sensitivity, such as SPS, may influence children’s neurocognitive responses to emotional stimuli—mechanisms that could represent early markers for later socio-affective maladjustment.

### 1.3. The Present Study

The present study aimed to examine how children’s tendencies towards social affiliation relate to their attention to emotional facial expressions, and how this relationship may vary depending on the level of SPS in their parents. Using eye tracking, we measured children’s visual attention to faces expressing happiness, sadness, fear, and anger. We were particularly interested in whether children who tend to avoid social engagement also display less attention to emotional cues, and whether this link is influenced by how sensitive their parents are to emotional stimuli. By exploring the interplay between child social tendencies and parental sensitivity, we hoped to gain a better understanding of the early factors that shape children’s emotional development. Based on the differential susceptibility framework, we hypothesized that parental Sensory Processing Sensitivity (SPS) contributes to the association between children’s social affiliation and their visual attention to emotionally salient (angry) faces. Specifically, we expected that the influence of social affiliation on attention to threat cues would vary depending on the degree of SPS in the parent.

## 2. Method

### 2.1. Participants

The study sample included 153 children (*Mage* = 9.97; *SDage* = 1.28; 48.8% females), and one of their parents who completed questionnaires for their children’s and their own behavior (N = 153; *Mage* = 40.9; *SDage* = 4.85; 87.7% females). Families were recruited through a screening process as part of a nationwide study conducted by our university, which involved 16,000 elementary school students. Children and their parents visited the local lab to participate in a passive-viewing eye-tracking computerized task. Ethical approval was granted by the Cyprus Bioethics Review Board (Approval No. 2019/73). Parents gave written informed consent for both their own and their child’s participation. Before taking part, families were briefed by the research team on the study’s nature and overall aims and were invited to participate in the experiment. Before the visit, parents completed an online questionnaire battery, using a secure internet-based platform (REDCap) to assess their own and their child’s sociodemographic, temperamental, and emotional–behavioral traits. Parents were also asked to report whether their child had a history of epilepsy or any other serious mental or physical handicap that could preclude their participation. None was reported. All participants were Greek Cypriots—the majority ethnolinguistic group in Cyprus—and demonstrated strong proficiency in the Greek language.

### 2.2. Assessment of Parental Behavior

The SPS instrument for parents was translated into Greek and then back-translated into English in accordance with the guidelines provided by the original standardization team [25].

Highly Sensitive Person Scale: Parental sensitivity was assessed using a brief 12-item version of the Highly Sensitive Person (HSP) scale, a shortened form of the original 27-item HSP scale, which maintains comparable psychometrics and constructs validity properties [25]. Each item was rated on a 7-point Likert scale, ranging from 1 = strongly disagree to 7 = strongly agree. The scale included items such as “I get nervous when I have to do a lot in little time”, “Some music can make me really happy”, and “Loud noises make me feel uncomfortable”. A total sensitivity score was calculated by averaging all item responses, with higher scores indicating greater sensitivity. The overall internal consistency of the scale was high (Cronbach’s α = 0.80).

### 2.3. Assessment of Child Behavior

Social Affiliation: The Affiliation Scale of the Early Adolescent Temperament Questionnaire is a 6-item scale designed to capture the desire for warmth and closeness with others [26]. Parents rated their children on a 5-point Likert scale, which ranges from “Does not apply at all” to “Applies very well” (α = 0.85). Parents completed the Affiliation Scale for their own child during the same online session in which they reported their own Sensory Processing Sensitivity (prior to the child’s lab visit). This timing ensured that parental ratings of child social affiliation (e.g., “My child enjoys cozying up to others”) were based on their child’s typical behaviors and unaffected by the experimental task.

### 2.4. Child Attentional Patterns

Children’s attentional patterns in response to emotional faces were measured using eye-tracking technology, which quantified the total fixation duration (in milliseconds) on predefined areas of interest (AOIs), such as the eyes and mouth regions of faces expressing happiness, sadness, fear, and anger. This objective measure provided a continuous scale of attentional engagement, allowing for an analysis of how children distribute their gaze across emotionally salient facial features.

### 2.5. Experimental Materials

**Figure 1:**
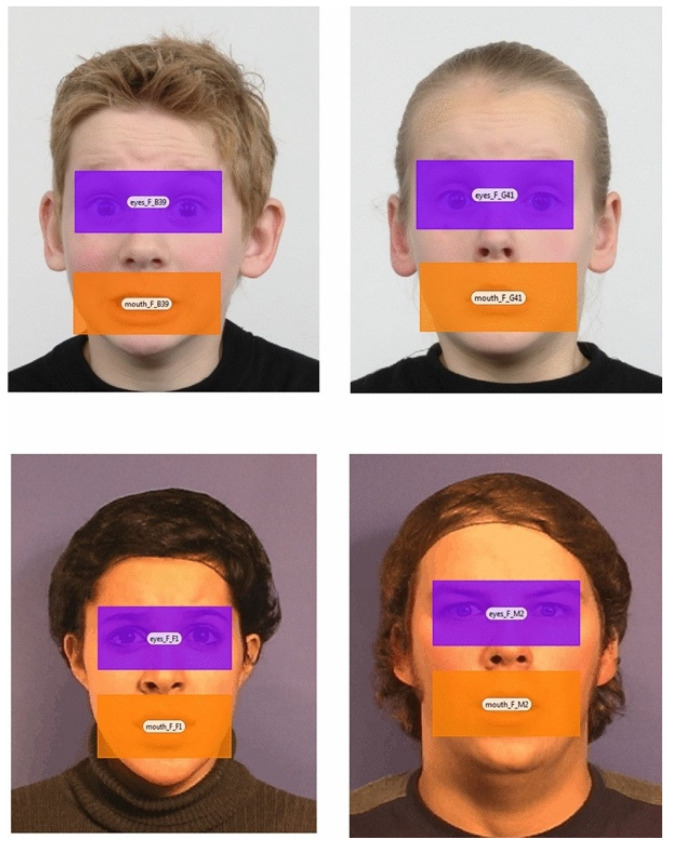
Areas of interest (AOIs) used in the emotional processing eye-tracking task—examples from the expression of fear in male/female and child/adult actors.

The eye-tracking experiment featured static, front-facing images of adult and child faces expressing four basic emotions: anger, sadness, fear, and happiness. These images were sourced from the Radboud Faces Database [27] and had been validated in prior studies with Cypriot children [28]. The stimulus set included expressions from five adult actors (50% female), with each actor displaying all four emotions across two snapshots (4 actors × 4 emotions × 2 snapshots). Additionally, four child actors were included, with 32 static images selected to match the emotional expressions found in the adult images. As part of the initial validation process in a previous child study conducted in our laboratory ([28]; see also [29,30]), participants categorized the static images into one of the four affective states and rated the intensity of the expressions (high vs. low) [28]. Based on this validation, the final experiment included 16 images of children and 16 images of adults. The 32 images were presented in a pseudo-randomized order to prevent sequential repetition (see Figure 1).

### 2.6. Apparatus

The eye-tracking experiment assessed the real-time attention allocation during an emotion-processing task using Tobii Pro Nano eye-tracking software Tobii Pro Studio 3.4.3 (Tobii Technology, Inc., Washington, DC, USA). The Tobii Pro Nano is a bright-pupil eye tracker equipped with a high-resolution camera and a wide field of view, capturing participants’ eye movements at a sampling rate of 60 Hz. The system employs two near-infrared diodes to illuminate the eyes, creating reflection patterns on the corneas. A high-resolution video camera records these reflections and calculates the participant’s gaze position on the screen based on the reflection pattern and their individual positioning. The stimuli were shown on a 22-inch computer monitor with a maximum resolution of 1680 × 1050 pixels. To ensure precise gaze tracking, each participant underwent a standard 5-point calibration procedure. A calibration test was conducted prior to the emotion-processing task to verify the recording accuracy. After successful calibration, participants proceeded with the task. The presentation timing of the visual stimuli and real-time gaze tracking were managed using Tobii Pro Studio 3.4.3, in accordance with the manufacturer’s guidelines (Tobii Studio User’s Manual 3.4.3). To test the study’s hypotheses, areas of interest (AOIs) were defined around the eyes, mouth, and whole face. However, for the present study, only the eye and mouth AOIs were analyzed. These AOIs were marked using two rectangular regions, aligned with the lower and upper and left and right boundaries of the eyes and mouth (see Figure 1). The predefined AOI sizes remained consistent across all facial emotional stimuli. Eye gaze data were analyzed by calculating the total dwell time within two predefined areas of interest (AOIs), namely, the eyes and mouth. The total fixation duration was computed separately for each AOI and for each emotional expression (e.g., fear, anger, sadness, and happiness). The recordings were processed offline using the automated Tobii Pro Lab eye-tracking software and Eye Tracker Manager, following the manufacturer’s manual instructions.

### 2.7. Experimental Procedure

Upon arriving at the lab, families were welcomed by a researcher who provided a detailed explanation of the consent form and answered any questions about the study procedures. Children’s assent was obtained before they participated in the experiment. After consent was signed, the children were given detailed information about the experimental process. Participants were subsequently guided to the main lab area, where they were seated in front of a desktop computer in a well-lit room. The children were seated comfortably in height-adjustable chairs, adjusted to ensure they could look directly at the screen, allowing for accurate gaze recording. The participants’ heads were positioned approximately 60 cm from the computer monitor. They were instructed to avoid moving their heads or covering their faces, and a 5-point calibration process was conducted. During this calibration, the children followed a green circle moving around the screen, repeating the procedure until high-accuracy gaze tracking was achieved. Emotional stimuli were displayed for three seconds, with pictures of adult and child actors shown alternately during the eye gaze condition. Each trial included (1) a one-second fixation cross in the center of the screen, followed by (2) a three-second presentation of a static facial expression. The task lasted approximately 15 to 20 min. After completing the experimental phase, the participants and their parents were debriefed about the study’s objectives.

### 2.8. Plan of Analyses

Initial exploratory analyses included Pearson/Spearman correlations between all continuous variables (child social affiliation, parental SPS, and total fixation duration for each emotion and facial feature). When normality assumptions for independent variables were not met, Spearman correlation analyses were conducted instead of Pearson correlations. Additionally, *t*-tests were computed to compare questionnaire scores between males and females. For our primary hypothesis testing, we conducted repeated-measure ANOVAs with a 4 (emotional valence: happy, sad, angry, fearful) × 2 (AOI: eyes vs. mouth) within-subject design, with two continuous between-subject factors, child social affiliation (z-scored) and parental SPS (z-scored; M = 4.32, SD = 1.14, range = 1.58–6.83 on 7-point scale), and child sex as a covariate. The total scores of the questionnaires were employed for this initial set of analyses, as they were deemed appropriate for this study’s objectives. This analytical approach allowed us to examine how children’s social affiliation relates to attention patterns, assess the contributing role of parental SPS to these attention patterns, and investigate whether parental SPS influences the relationship between child social affiliation and attention.

Effect sizes (eta squared) were reported, with values <0.01 considered small, values ≤0.06 considered medium, and values ≥0.14 considered large. Significant interactions were illustrated in the figures, accompanied by standard errors. The Greenhouse–Geisser correction was applied to adjust the degrees of freedom for the F-ratios, and post hoc pairwise comparisons were corrected using the Bonferroni–Holm method. Interaction effects involving social affiliation and parental SPS were examined to explore whether parental SPS contributed to the relationship between children’s socio-affiliative tendencies and attention to emotional faces. Significant interactions were further probed through post hoc visualizations using an open-source interactive tool that generated small multiple plots (individual plots for each level of the moderator) and marginal effect plots, enabling a clearer interpretation of regions of significance and potential moderation patterns across levels of parental SPS. All analyses were conducted using SPSS 29 (IBM Statistics, 2022, Armonk, NY, USA: IBM Corp).

## 3. Results

### 3.1. Descriptive Statistics

Independent *t*-tests were conducted to compare the questionnaire scores between the male and female participants. These analyses revealed no significant sex differences for social affiliation, parental SPS, or fixation measures across the emotional face regions.

### 3.2. Behavior by Eye Gaze Correlations

Pearson and Spearman correlations between the child and parent SPS, child rates of social affiliation, and eye gaze data did not reveal any significant correlations.

### 3.3. Behavior by Eye Gaze Associations

**Figure 2:**
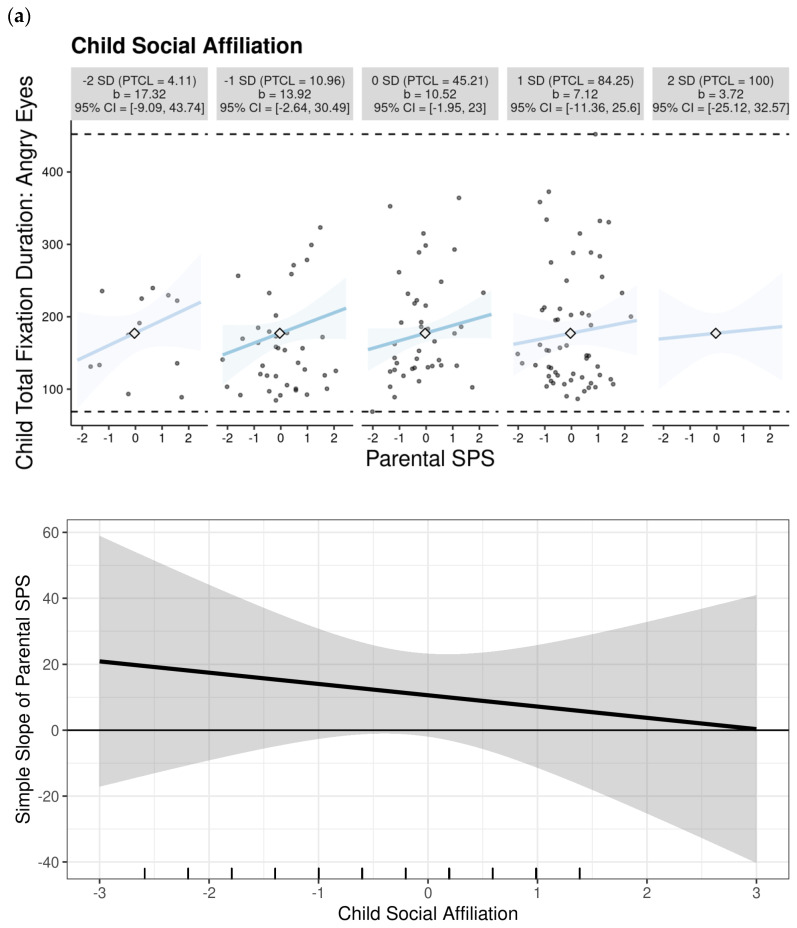

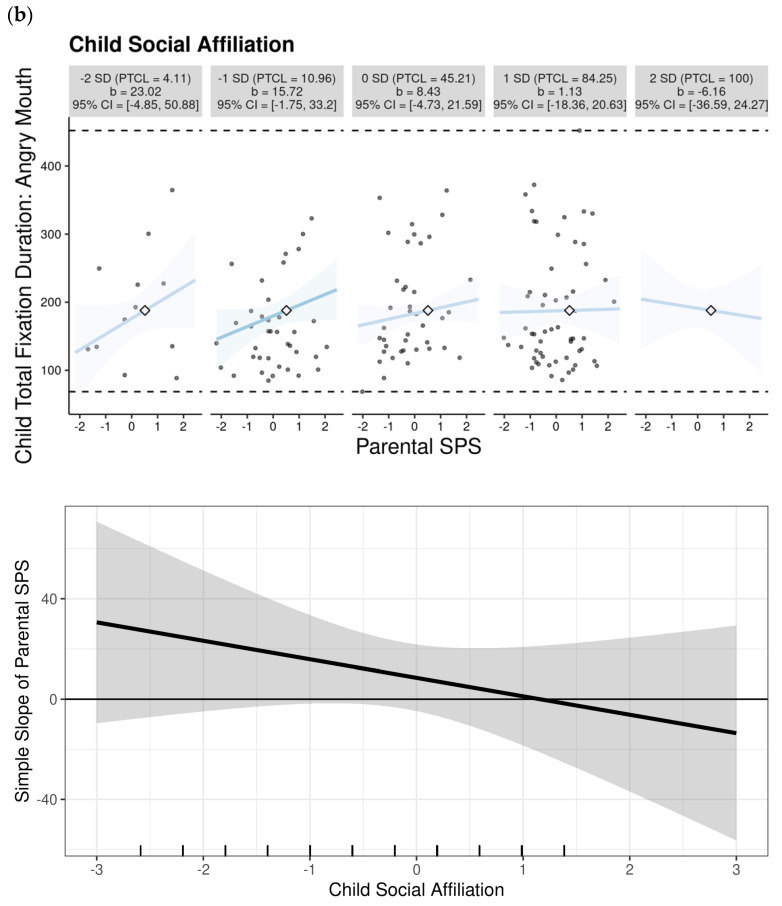
Conditional relationships between child social affiliation and attention to (**a**) angry eyes and (**b**) angry mouth regions, across different levels of parental Sensory Processing Sensitivity (SPS). Small multiple plots show the association at quintiles of parental SPS (from lowest 20% to highest 20%) to illustrate how the patterns vary across the sensitivity spectrum. Marginal effect plots (right panels) display the continuous interaction with 95% confidence bands. SD: standard deviation. Each simple slope includes the computed 95% confidence region and percentile, PTCL, as recommended by McCabe et al. [31].

A four (emotional valence: happy versus sad, angry, or fearful) by two (AOIs: mouth versus eyes) mixed analysis of variance (ANOVA) with two continuous between-subject factors, child social affiliation rates and parental SPS scores, and child sex as a covariate was conducted. The analysis revealed a three-way AOI by social affiliation x parental SPS effect [F (23, 198) = 3.47, η^2^ = 0.90, *p* < 0.001], where participants with higher rates of social affiliation and parental SPS exhibited, in general, higher total fixation durations towards the eye regions of the emotional faces (see Figure 2, plots a–b). The analysis revealed that parental SPS contributed to the association between social affiliation and attention patterns, as evidenced by the significant four-way emotion by AOI by affiliation by parental SPS rate effect [F (23, 198) = 1.96, η^2^ = 0.85, *p* < 0.001], where participants with higher rates of social affiliation and parental SPS had significantly higher total fixation durations towards the mouth regions of angry faces (see Figure 2, plots a–b). The interaction effects are visualized in Figure 2 using two complementary approaches: (1) small multiple plots showing the social affiliation–attention relationship at different levels of parental SPS (quintiles), and (2) continuous marginal effect plots. This dual presentation demonstrates that children with lower social affiliation showed heightened attention to angry mouths primarily when parents had higher SPS (top 40% of scores), while this pattern was absent at lower SPS levels. No additional main or interaction effects were evident.

## 4. Discussion

The significant effect of eye movements towards the angry mouth region on the child social affiliation rates, with parental SPS as a contributing factor, underscores the intricate relationship between parental traits, child behaviors, and social interactions. The findings highlight that children’s social responses to facial emotional stimuli of anger are influenced by parental SPS levels, indicating a broader impact on their social development and emotional processing. Our results link to the evidence suggesting that SPS parents may influence children’s emotional development by becoming more overwhelmed by their parenting responsibilities and adopting more harsh parenting behavior [22]. In sum, the results unveil the possibility that higher parental SPS may lead to the increased desensitization of children to cues of threats or negative reinforcement, especially when accompanied by lower social affiliation (e.g., [13,14]), and in our study, this is represented by the increased total fixations towards the angry mouth regions of faces early in life.

In particular, as given on the multiple small plots and marginal effect plots, the study’s data revealed that eye movements towards these specific AOIs had the most significant effect on the child social affiliation rates when considering the contributing influence of parental SPS levels. Specifically, the results indicated that the interaction between the parental SPS rates and child social affiliation rates was most pronounced when the children exhibited increased eye movements towards the angry mouth regions. This finding suggests that parental SPS levels may play a crucial role in shaping how children respond socially to emotional cues early in life. Children with higher social affiliation rates may exhibit different patterns of expressing anger compared to children with lower social affiliation rates. Also, parents with varying levels of SPS rates may perceive and respond to their children’s anger expressions differently. The current pattern of findings aligns with the hypothesis that parents with higher sensitivity levels may be more attuned to subtle cues and emotional signals, affecting how they interpret and react to their child’s anger, and this association may be influenced by the child’s social affiliation rates. As our observations are solely based on a laboratory study, this hypothesis requires further comprehensive investigation by accounting for additional variables that are associated with parenting style [14] and child–parent bonding early in life [4,5].

Parents with higher SPS levels may exhibit a heightened sensitivity to emotional cues, which could shape their children’s social behaviors and responses to different emotional expressions. The observed contributing effect suggests that parental traits play a pivotal role in guiding children’s social affiliations, particularly in response to expressions of anger. Understanding how parental SPS influences the relationship between child social affiliation rates and eye movements towards these specific emotional cues provides valuable insights into the complexities of social development in children. From the viewpoint of the early manifestations of CU trait-related temperament characteristics, the study’s pattern of findings is aligned with the hypothesis that parenting can be counted as an important environmental factor for the development of CU traits, while the temperament characteristic of social affiliation may undermine the parent–child relationship, which can lead to even harsher and less warm parenting, in a bidirectional manner (for a review, see [6]). This hypothesis requires further investigation by conducting multi-level longitudinal investigations of such bidirectional mechanisms.

The current findings are informed by theoretical frameworks that position CU traits as an important developmental construct associated with reduced social affiliation, atypical affective processing, and diminished responsiveness to social–emotional cues. In this context, low affiliative tendencies in childhood—as examined in the present study—can be understood as part of a broader continuum of social–emotional functioning relevant to CU traits. Our findings, particularly the differential attention to angry faces as a function of child affiliative behavior and parental SPS, align with models that propose early divergence in social motivation and emotional attention as potential markers in CU pathways. While CU traits were not directly assessed, the affiliative construct examined here reflects a theoretically meaningful correlate that helps extend our understanding of how early parent–child dynamics might shape individual differences in children’s social information processing.

In addition, a methodological limitation in many emotion-processing studies in children and adults concerns the use of static images of emotional expressions to measure attention allocation. Static images may not fully capture the dynamic nature of emotions, which unfold over time in real-life experiences. However, for the needs of the present study, we chose to employ static pictures to allow for more safe future comparisons among different groups of participants in different developmental stages.

Despite these limitations, the present study provides novel insights into the potential contribution of parental environmental sensitivity in shaping early emotional processing in children with temperamental characteristics that may lead to CU traits. Future research endeavors could delve deeper into the underlying mechanisms driving these effects and explore additional factors that may influence the interplay between parental SPS, child social affiliation rates, and eye movements towards various emotional expressions. The association between child social affiliation rates, parental SPS rates, and emotional processing can impact the emotional support available within the family. Understanding how these factors interact can shed light on how parents create a safe space for their children to express aggression, process emotions, and seek comfort during times of distress. By unraveling these dynamics, researchers can contribute to a more nuanced understanding of how parental characteristics impact children’s social behaviors and emotional responses, paving the way for tailored interventions and support strategies aimed at fostering healthy social development in children. This analysis contributes to the broader understanding of how parental factors may shape and influence the social development and responses of children in various contexts.

Although the current study employed repeated-measure ANOVAs rather than regression-based moderation models, we conceptualized parental Sensory Processing Sensitivity (SPS) as an individual difference factor that contributes to the variation in children’s attentional responses to emotional faces. The interaction effects observed in the ANOVAs suggest that SPS plays a role in shaping the social–emotional environment in which child affiliative tendencies relate to their attention to angry faces. To further explore these interaction effects, we used open-source interactive data visualization tools that allowed us to inspect patterns across levels of parental SPS. These visualizations highlighted differences in attentional engagement depending on parental SPS levels, providing insight into how such characteristics may influence children’s social information processing.

The current findings highlight an important, previously underexplored pathway by which parental environmental sensitivity may influence early emotional processing in children. This interaction has meaningful implications for both theory and practice. Theoretically, it expands developmental models of social affiliation and CU traits by positioning parental SPS not just as a background trait but as a potential factor of risk processes in children. Practically, these findings point to the value of including parental SPS in risk assessment and intervention planning. Identifying families with high-SPS parents and children with low social affiliation could help clinicians design targeted parent–child interventions that emphasize emotional regulation, responsive parenting, and attunement strategies. For instance, psychoeducational programs could be developed to help highly sensitive parents understand the impact of their own emotional reactivity on their child’s perception and processing of affective stimuli.

Moreover, this study’s findings could inform preventive approaches within school and clinical settings by integrating SPS profiles into broader socioemotional screening tools. This would allow for the earlier identification of children who may be at risk for difficulties in emotional understanding or later socio-behavioral maladjustment. Future research should explore whether modifying parental emotional awareness or arousal regulation—particularly in high-SPS parents—could mitigate children’s attention to negative emotional cues and enhance their affiliative behaviors. Ultimately, this study contributes to a more nuanced understanding of how individual differences within the parent–child dyad interact to shape foundational aspects of social cognition and emotional development.

## Data Availability

The datasets generated and/or analyzed during the current study are available from the corresponding author upon reasonable request due to privacy.
